# Machine Learning Predictors of Extreme Events Occurring in Complex Dynamical Systems

**DOI:** 10.3390/e21100925

**Published:** 2019-09-23

**Authors:** Stephen Guth, Themistoklis P. Sapsis

**Affiliations:** Department of Mechanical Engineering, Massachusetts Institute of Technology, 77 Massachusetts Ave., Cambridge, MA 02139, USA; sguth@mit.edu

**Keywords:** optimal predictors, binary classification, rare extreme events, chaotic systems, data-driven methods

## Abstract

The ability to characterize and predict extreme events is a vital topic in fields ranging from finance to ocean engineering. Typically, the most-extreme events are also the most-rare, and it is this property that makes data collection and direct simulation challenging. We consider the problem of deriving optimal predictors of extremes directly from data characterizing a complex system, by formulating the problem in the context of binary classification. Specifically, we assume that a training dataset consists of: (i) indicator time series specifying on whether or not an extreme event occurs; and (ii) observables time series, which are employed to formulate efficient predictors. We employ and assess standard binary classification criteria for the selection of optimal predictors, such as total and balanced error and area under the curve, in the context of extreme event prediction. For physical systems for which there is sufficient separation between the extreme and regular events, i.e., extremes are distinguishably larger compared with regular events, we prove the existence of optimal extreme event thresholds that lead to efficient predictors. Moreover, motivated by the special character of extreme events, i.e., the very low rate of occurrence, we formulate a new objective function for the selection of predictors. This objective is constructed from the same principles as receiver operating characteristic curves, and exhibits a geometric connection to the regime separation property. We demonstrate the application of the new selection criterion to the advance prediction of intermittent extreme events in two challenging complex systems: the Majda–McLaughlin–Tabak model, a 1D nonlinear, dispersive wave model, and the 2D Kolmogorov flow model, which exhibits extreme dissipation events.

## 1. Introduction

Many phenomena in a wide range of physical domains and engineering applications have observable properties that are normally distributed, that is, they obey Gaussian statistics. Gaussian-distributed random variables and processes are particularly easy to manipulate algebraically, and there is a rich literature using their properties in widely varying areas of probability and statistics, from Bayesian regression [1] to stochastic differential equations [2]. In many applications, however, random variables have significantly non-Gaussian character. Frequently, the “long-tails” of their distribution, which contain extreme, but rare events, are particularly important for a complete understanding of the phenomena in question. Examples of this behavior occur in systems ranging from ocean waves [3,4] to finance [5], but similar phenomena are observed in fields as far afoot as cell dynamics [6], mechanical part failure [7], and turbine shocks [8].

Several approaches have been developed that successfully resolve the statistics capturing extreme events (see, e.g., [9,10,11,12,13,14]). These often take advantage of the special structure of the problem, which is of course the preferred approach in cases where the dynamics are well understood. For systems where our understanding of the governing laws is partial or in situations where there are important model errors, it can be shown that combining the available imperfect models with data-driven ideas can result in prediction schemes that are more effective than each of the ingredients, data or imperfect model, used independently [15]. Another perspective, for systems where we can control which samples to use or compute, is to apply optimal experimental design ideas or active learning [16]. In this case, one can optimize the samples that should be used, using information from samples already available, in order to have accurate statistics with a very small computational or experimental cost.

There are situations, however, where the only information available that characterizes the system is observations—“big data”. For such cases, one important class of problems is to identify extreme event precursors—system states that are likely to evolve into extreme events. Successful identification of precursors requires both a careful definition of what exactly qualifies as an extreme event, as well as a balance between false positive and false negative errors [17,18,19,20,21,22]. While there is already a vast literature in machine learning related to binary classification, little is specifically directed to the problem of extreme event precursors and prediction.

In this paper, we first discuss limitations of standard methods from binary classification, in the context of extreme event precursors. Motivated by these limitations, we design a machine learning approach to compute optimal precursors to extreme events (“predictors”) directly from data, taking into account the most important aspect of extreme events: their rare character. This approach will naturally suggest a frontier for trade-offs between false positive and negative error rates, and in many cases will geometrically identify an optimal threshold to separate extreme and quiescent events. We demonstrate the derived ideas to two prototype systems that display highly complex chaotic behavior with elements of extreme, rare events: the Majda–McLaughlin–Tabak model [23] and the Kolmogorov flow [24]. In the first system, we compare our method to a standard loss function (total error rate) and show how it leads to better predictors. In both systems, machine-learned predictors support previous analysis on the mechanisms of intermittency in those systems.

## 2. A Critical Overview of Binary Classification Methods

The problem of identifying good precursors for extreme events can also be seen as a binary classification problem. In this case, a training dataset takes the form of a set of pairs $S=\{\left(a_{i},x_{i}\right)\}$, where $a_{i}$ is a scalar indicator or quantity of interest at time $t_{i}$, whose value defines whether or not we have an extreme event. The vector $x_{i}$, on the other hand, contains all the possible observables available up to time $t_{i}-\tau$ that we can utilize to predict whether we have an extreme event or not in the near future, i.e., after time τ. The aim is to identify the most effective function b=b(x) so that using the value of *b* we can predict if *a* will exhibit extreme behavior.

For any given function b(x), the set of these predictor–indicators pairs together defines a two dimensional joint distribution, with probability density function (pdf) $f_{ab}\left(a,b\right)$ and cumulative distribution function (cdf)

(1) $$F_{ab}\left(\hat{a},\hat{b}\right)=P\left(a<\hat{a},b<\hat{b}\right).$$

We use hat notation when the choice of $\hat{a}$ or $\hat{b}$ corresponds to a threshold value. A value exceeding the threshold is called extreme, and a value not exceeding it is called quiescent. The joint distribution may be constructed from simulations, from experimental measurements, or from analytical models. We use the term *histogram* in this work to refer to the data and their functional representation as a probability distribution.

**Figure 1:**
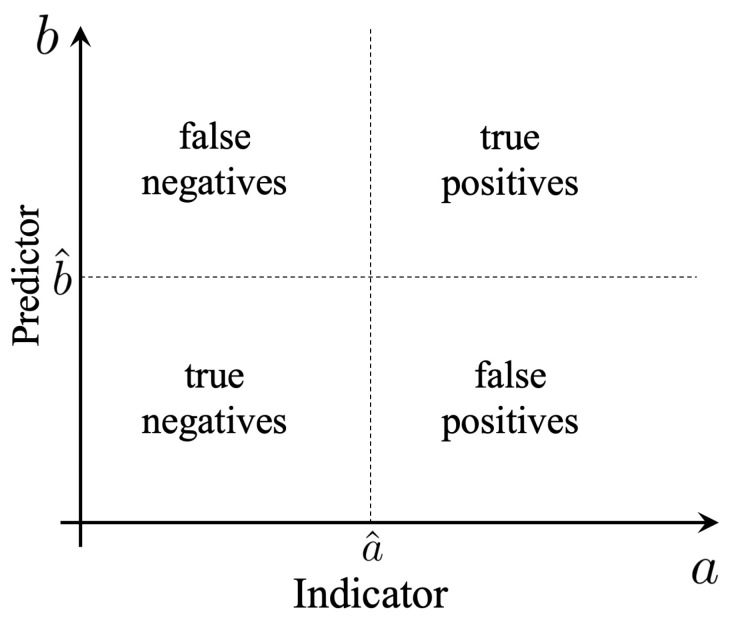
Optimization of predictors as a binary classification problem.

A fixed choice of $\hat{a}$ and $\hat{b}$ defines a binary classification problem with four possibilities (Figure 1):

- True Positive (TP): an event predicted to be extreme $\left(b>\hat{b}\right)$ that is actually extreme $\left(a>\hat{a}\right)$;
- True Negative (TN): an event predicted to be quiescent $\left(b<\hat{b}\right)$ that is actually quiescent $\left(a<\hat{a}\right)$;
- False Positive (FP): an event predicted to be extreme $\left(b>\hat{b}\right)$ that is actually quiescent $\left(a<\hat{a}\right)$;
- False Negative (FN): an event predicted to be quiescent $\left(b<\hat{b}\right)$ that is actually extreme $\left(a>\hat{a}\right)$.

### 2.1. Total and Balanced Error Rate

Based on this classification, we can define several criteria for the selection of predictors. The typical binary classification task minimizes the *total error rate*, which is defined as

(2) $$E_{T}=P\left(FP\right)+P\left(FN\right)=P\left(a<\hat{a},b>\hat{b}\right)+P\left(a>\hat{a},b<\hat{b}\right)$$

This error metric is poorly suited for the extreme event prediction problem for two reasons:

- First, total error rate is unsuited for unbalanced data. Extreme events are usually associated with extremely unbalanced datasets. This manifests in two ways. First, even a naive predictor may achieve very high accuracy, simply because always predicting “not extreme” is usually correct. Second, resampling the data (for instance, to balance the number of extreme and not-extreme training points) may widely change the total error rate, which in turn may change the optimal predictor.
- Second, this error metric is unsuited for strength-of-confidence measurement. It has no ability to distinguish between confidently classified points and and un-confidently classified points. This is particularly important if we expect our predictor to make many mistakes.

The first objection may be resolved by using balanced error quantities, such as the balanced error rate:

(3) $$E_{B}=1-\frac{1}{2}\left(\frac{P(TP)}{P(TP)+P(FN)}+\frac{P(TN)}{P(TN)+P(FP)}\right),$$

which measures the true-positives and true-negatives but normalized by the number of predicted positives and negatives, respectively.

### 2.2. $F_{1}-$Score

Another criterion to deal with the strongly unbalanced character of the datasets that contain extreme rare events is the $F_{1}-$score. We first define several other important quantities that we use and study their basic properties.

In particular, precision, denoted by *s*, is the probability that an event is a true positive, given that it is predicted to be extreme:

(4) $$s\left(\hat{a},\hat{b}\right)=\frac{P(TP)}{P(TP)+P(FP)}=\frac{1+F_{ab}\left(\hat{a},\hat{b}\right)-F_{a}\left(\hat{a}\right)-F_{b}\left(\hat{b}\right)}{1-F_{b}\left(\hat{b}\right)}\;\;[\mathrm{Precision}].$$

The recall (sometimes called sensitivity), denoted by *r*, is the probability that an event is a true positive, given that is actually extreme:

(5) $$r\left(\hat{a},\hat{b}\right)=\frac{P(TP)}{P(TP)+P(FN)}=\frac{1+F_{ab}\left(\hat{a},\hat{b}\right)-F_{a}\left(\hat{a}\right)-F_{b}\left(\hat{b}\right)}{1-F_{a}\left(\hat{a}\right)}\;\;[\mathrm{Recall}],$$

Furthermore, the extreme event rate, denoted by *q*, is the probability that an event is actually extreme:

$$\begin{array}{cc} q(\hat{a}) & =P\left(TP\right)+P\left(FN\right)=P\left(a>\hat{a}\right)=1-F_{a}\left(\hat{a}\right)\;\;[\mathrm{Extreme}\;\mathrm{event}\;\mathrm{rate}]. \end{array}$$

By construction, the derived quantities have the following monotonicity properties:

**Theorem** **1** (Monotonicity)**.** *The precision function, $s(\hat{a},\hat{b})$, is monotonic in its first argument, $\hat{a}$, while the recall function, $r(\hat{a},\hat{b})$, is monotonic in its second argument, $\hat{b}$. Furthermore, the extreme event rate, $q(\hat{a})$, is a monotonic function with respect to its argument, $\hat{a}$.*

Additionally, the choice of precision and recall as binary classification metrics is motivated by the following three invariance properties

**Theorem** **2** (Invariance-I)**.** *Precision, recall, and extreme event rate are entirely determined by the function $F_{ab}$, and the thresholds $\hat{a}$ and $\hat{b}$.*

**Theorem** **3** (Invariance-II)**.** Let $f_{1}\left(a,b\right)$ and $f_{2}\left(a,b\right)$ be two histograms with the property that, outside of a certain region $\left[-\infty ,a^{*}\right]⊗\left[-\infty ,b^{*}\right]$, $f_{1}\left(a,b\right)=\beta f_{2}\left(a,b\right)$ for some fixed constant β. That is to say, $f_{1}\left(a,b\right)$ and $f_{2}\left(a,b\right)$ correspond to histograms that differ only by some number of points $\left(a_{i},b_{i}\right)$ for which $a_{i}<a^{*}$ and $b_{i}<b^{*}$.

Then, for all $\hat{a}>a^{*}$ and $\hat{b}>b^{*}$, $s\left(\hat{a},\hat{b};F_{1}\right)=s\left(\hat{a},\hat{b};F_{2}\right)$ and $r\left(\hat{a},\hat{b};F_{1}\right)=r\left(\hat{a},\hat{b};F_{2}\right)$.

**Theorem** **4** (Invariance-III)**.** *Let $h_{1}\left(y\right),h_{2}\left(y\right):R\to R$ be order-preserving monotonic functions. Let $F_{ab}$ be a histogram, and let $F_{a^{\prime }b^{\prime }}$ be the histogram formed from the dataset $\left\{\left(h_{1}\left(a_{i}\right),h_{2}\left(b_{i}\right)\right)\right\}$. Then,*

(6) $$\begin{array}{cc} s(\hat{a},\hat{b};F_{ab}) & =s(h_{1}\left(\hat{a}\right),h_{2}\left(\hat{b}\right);F_{a^{\prime }b^{\prime }})\\ r(\hat{a},\hat{b};F_{ab}) & =r(h_{1}\left(\hat{a}\right),h_{2}\left(\hat{b}\right);F_{a^{\prime }b^{\prime }})\\ q(\hat{a};F_{ab}) & =q(h_{1}\left(\hat{a}\right);F_{a^{\prime }b^{\prime }}) \end{array}$$

*In other words, precision, recall, and extreme event rate are invariant under arbitrary nonlinear monotonic rescalings of the indicator and predictor.*

**Proof.**  The proof of Theorem 2 follows directly from the definitions of *q*, *r*, and *s* in Equation (6), where they are expressed in terms of conditional probabilities.

The proof of Theorem 3 follows from the restatement of the definition in Equation (6) in the form of ratios of cumulative density functions. The numerators can be written in cumulative distribution form as $1+F_{ab}\left(\hat{a},\hat{b}\right)-F_{a}\left(\hat{a}\right)-F_{b}\left(\hat{b}\right)=\int_{\hat{b}}^{\infty }\int_{\hat{a}}^{\infty }f_{ab}\left(a,b\right)dadb$, which takes as support $f_{ab}$ only in the complement of the region $\left[-\infty ,a^{*}\right]⊗\left[-\infty ,b^{*}\right]$. Similarly, the denominator support does not intersect the variable region. Because *s* and *r* are ratios of definite integrals, each of which is linear in its integrand, they are invariant under the linear rescaling β. Note that this is not true for *q*, which is not defined as a ratio.

Finally, the proof of Theorem 4 follows from the familiar *u*-substitution rule of ordinary calculus, restricted to the well behaved class of monotonic substitutions. □

These invariance properties simplify the issue of scale in the choice of predictor functions, limiting the hypothesis space of potential predictors. Using the precision and recall function, we define the the F-*score*, given by the harmonic mean of the precision and the recall:

(7) $$F_{1}=\frac{2}{r^{-1}+s^{-1}}.$$

Both the $F_{1}-$score and the balanced error depend on normalized quantities which take into account the extremely unbalanced character of the datasets. However, their value depends on the thresholds $\hat{a}$ and $\hat{b}$.

### 2.3. Area under the Precision–Recall Curve

To overcome the dependence on the $\hat{b}-$threshold value, we we consider a fixed extreme event rate, $q(\hat{a})$, or equivalently fixed $\hat{a}$, and define the *precision–recall curve* (sr curve) as

(8) $$\rho \left(\hat{b};\hat{a}\right)=\left(r\left(\hat{a},\hat{b}\right),s\left(\hat{a},\hat{b}\right)\right)$$

Because $r(\hat{a},\hat{b})$ is invertible in its second argument (Theorem 1), this curve gives precision as a unique function of recall and the extreme event rate:

(9) s=s(r;q)

**Figure 2:**
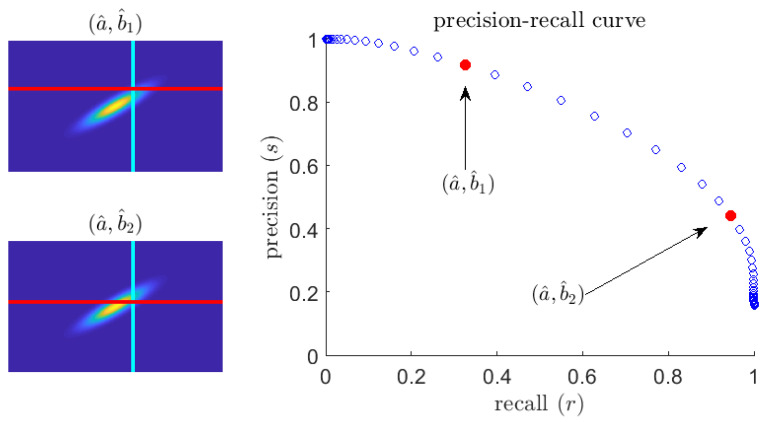
(**left**) Plot of a sample pdf $f_{ab}$; (**right**) the corresponding precision–recall sr curve. The parameterized curve is generated by fixing $\hat{a}$ and letting $\hat{b}$ vary.

An example sr curve (for fixed *q*) is exhibited in Figure 2. Smaller values of recall correspond to larger values of precision and vice versa. This is intuitive: to be sure to catch every extreme event (high recall), the predictor will have to let through many false positive quiescent events (low precision). Note the precision does not vanish as *r* increases, even when the predictor threshold, $\hat{b}$, is arbitrarily small. Instead, if that is the case, all events will be predicted as extremes and therefore the precision of the predictor will be given by the rate of extreme events. Therefore, the following limit holds:

**Theorem** **5** (Extreme Event Rate Correspondence)**.** *Let the sr curve, ρ, correspond to extreme event rate q. Then, we have the following limit*

(10) $$\lim_{r\to 1}s\left(r;q\right)=q$$

The sr curve is a variation of the Receiver Operating Characteristic (ROC) curve used in medical literature [19], which displays predictor performance over a range of threshold values. Here, we employ the sr curve as various reviews (see, e.g., [18]) have shown that it performs better on discriminating between classifiers when the data are wildly unbalanced. To obtain a metric that does not depend on a specific threshold value of the predictor, $\hat{b}$, a standard metric is the area under the curve (AUC), α.

In particular, for a fixed value of the rate *q* (equivalent to a fixed extreme event threshold $\hat{a}$), the area under the curve, α, is given by

(11) $$\begin{array}{cc} \alpha (q) & =\int_{0}^{1}s\left(r\right)dr=\int_{-\infty }^{\infty }s\left(\hat{b}\right)\left|\frac{\partial r}{\partial \hat{b}}\right|d\hat{b}. \end{array}$$

A larger value of α corresponds to a more favorable set of choices $\hat{b}$ that maximize both precision and recall in combination. The ideal rs curve would run from (0,1) to (1,1), and then down from (1,1) to (1,q). Therefore, we can choose a predictor by maximizing α for a fixed value of *q*. The value of *q*, however, has to be chosen in an ad-hoc manner and this motivates the introduction of the next measure.

### 2.4. Volume under the Precision–Recall–Rate Surface

To overcome the dependence on the ad-hoc parameter, $\hat{a}$, we generalize the notion of the precision–recall curve to the precision–recall–rate surface (qrs surface). This is the parametric surface defined by

(12) $$\sigma \left(\hat{a},\hat{b}\right)=\left(q\left(\hat{a}\right),r\left(\hat{a},\hat{b}\right),s\left(\hat{a},\hat{b}\right)\right).$$

**Figure 3:**
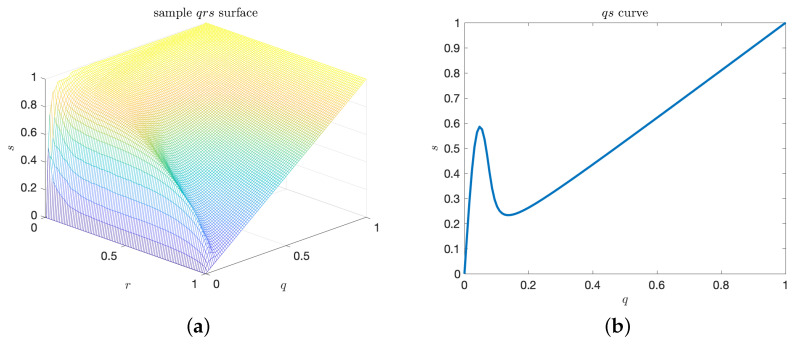
(**a**) Plot of a sample precision–recall–extreme-event-rate, qrs surface. The surface is generated by varying $\hat{a}$ and $\hat{b}$; (**b**) Precision–rate slice of the qrs plot, where r=0.5.

Such qrs surface is shown in Figure 3a. Similar to the sr curve, *q* and *r* may be inverted sequentially. By analogy with the area under the curve, α, we can define an enclosed volume functional for the qrs surface as well. In particular, we have the *volume under the surface*, *V*, given by

(13) $$\begin{array}{cc} V & =\int_{0}^{1}\int_{0}^{1}s\left(r,q\right)drdq. \end{array}$$

While *V* evaluates the goodness of a predictor over all possible pairs $\left(\hat{a},\hat{b}\right)$, it does not specifically quantify the quality of the predictor for extreme-events, i.e., low values of the rate, *q*.

## 3. Separation of Extreme and Quiescent Events

**Figure 4:**
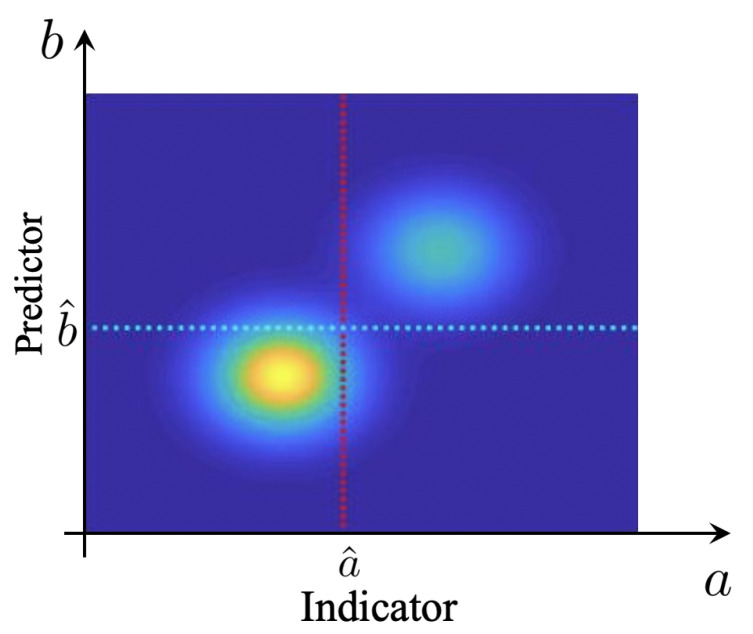
Sample pdf exhibiting separation of extremes from quiescent events, corresponding to the qrs surface in Figure 3a.

Before we proceed to the definition of a measure that explicitly takes into account the rare event character (or low rate) of extreme events, it is important to study some properties of the precision–recall–rate surface. We are interested in understanding the structure of these surfaces and the implications on selecting predictors, for physical systems exhibiting extreme events. For a large class of such systems, the causal mechanism of extremes are internal instabilities, combined with the intrinsic stochasticity of the system (see, e.g., [14,20,25,26,27]). In this case, the extreme events are distinguishably larger compared with regular events. This is because extreme event properties are primarily controlled by the system nonlinearity and the subsequent instabilities. For such cases, the joint pdf between indicator and predictor has a special structure where two distinguished probability regions can be observed. A sample pdf exhibiting this property is shown in Figure 4.

We have observed that this separation of regimes in the probabilistic sense implies interesting properties for the qrs surface (shown in Figure 3 for the pdf shown in Figure 4). In particular, the separation in the pdf $f_{ab}$ results in a knuckle point on the qs curves (Figure 3b). This knuckle point is associated with a local maximum of the precision for the particular choice of the rate, *q*. It essentially defines an optimal value of the extreme event threshold, $\hat{a}$, which, if chosen, optimizes the performance of the predictor.

Motivated by these observations, we study further the topological properties of the qrs surface. We have already presented several properties of the rs curves and to this end we focus on the qs curves. We first note the following basic properties:

**Theorem** **6** (Basic qs curve properties)**.** *Let $f_{ab}$ be a continuous joint pdf for the indicator and predictor, with finite support. Then, for all values of recall, r, the qs curve has the following properties:*

*1.*  *0≤s(q,r)≤1, for all q,r;*
*2.*  *s(q=0)=0 and s(q=1)=1; and*
*3.*  *$\frac{\partial s}{\partial q}\left(q=0\right)\geq 0$ and $\frac{\partial s}{\partial q}\left(q=1\right)\geq 0.$*

**Proof.**  Property 1 follows from the cdf form of Definition 4, which is expressed as a ratio. Both numerator and denominator are necessarily positive, leading to 0≤s(q,r). Furthermore, the numerator is written as the denominator minus a positive function, thus the numerator is never larger than the denominator, leading to s(q,r)≤1.

Property 2 follows from the confusion matrix formulation of Definition 4. When q=0, there are no true positive events, thus the numerator of *s* is identically zero. When q=1, there are no false positive events, so the numerator and denominator are equal.

Property 3 follows from the requirements of previous two properties and continuity. □

The above properties restrict the possible shapes of the qs curves. We are interested to understand conditions that lead to the occurrence of a knuckle point. Clearly, this is going to be the case when we have an interval of rates for which $\frac{\partial s}{\partial q}<0$. We compute this condition in terms of the joint pdf $f_{ab}$. We have the derivative of *s* with respect to *q* for constant *r*:

(14) $${\left.\frac{ds}{dq}\right|}_{r}=\frac{1}{\frac{\partial q}{\partial \hat{a}}\frac{\partial r}{\partial \hat{b}}}\left(\frac{\partial s}{\partial \hat{a}}\frac{\partial r}{\partial \hat{b}}-\frac{\partial s}{\partial \hat{b}}\frac{\partial r}{\partial \hat{a}}\right).$$

Substituting the expressions for the precision and recall function, we find that the sign of the above derivative is given by the function

(15) $$\begin{array}{cc} Q(\hat{a},\hat{b}) & =\int_{\hat{a}}^{\infty }\int_{-\infty }^{\infty }f_{ab}\left(a,b\right)dbda\int_{\hat{b}}^{\infty }f_{ab}\left(\hat{a},b\right)db\int_{-\infty }^{\infty }f_{ab}\left(a,\hat{b}\right)da\\ \; & +\int_{-\infty }^{\infty }\int_{\hat{b}}^{\infty }f_{ab}\left(a,b\right)dbda\int_{-\infty }^{\infty }f_{ab}\left(\hat{a},b\right)db\int_{\hat{a}}^{\infty }f_{ab}\left(a,\hat{b}\right)da\\ \; & -\int_{\hat{a}}^{\infty }\int_{\hat{b}}^{\infty }f_{ab}\left(a,b\right)dbda\int_{-\infty }^{\infty }f_{ab}\left(\hat{a},b\right)db\int_{-\infty }^{\infty }f_{ab}\left(a,\hat{b}\right)da \end{array}$$

The first two terms of *Q* will always be positive. That implies that *Q* can only change sign when the third term grows large enough in magnitude to dominate the other two terms. It is straightforward to show that this will be the case if there are threshold values such that the last term is larger than both the first and second term, i.e., the following sufficient condition holds for some threshold values:

(16) $$\begin{array}{c} s\left(\hat{a},\hat{b}\right)>P\left(b>\hat{b}|a=\hat{a}\right)\;\;\;and\;\;\;r\left(\hat{a},\hat{b}\right)>P\left(a>\hat{a}|b=\hat{b}\right) \end{array}$$

The first condition requires that the ratio of the true positives, P(TP), over all extremes, P(TP)+P(FP), is larger than the the conditional probability for a positive prediction $\left(b>\hat{b}\right)$ given that the indicator is just going to reach the threshold value, $\hat{a}$. The second condition requires that the ratio of the true positives, P(TP), over all extreme predictions, P(TP)+P(FN), is larger than the the conditional probability for an extreme $\left(a>\hat{a}\right)$ given that the predictor is just reaching the threshold value, $\hat{b}$. If both of these conditions hold, then there is an optimal value of $\hat{a}$ or *q* for which the precision of the predictor is optimized.

## 4. A Predictor Selection Criterion Adjusted for Extreme Events

We introduce a new criterion for optimizing predictors, which takes into account the special features of extreme events, in particular their low rate of occurrence. This criterion should also be able to distinguish predictors that optimally capture extreme events for the case where we have well separated extreme events (knuckle point in the qs curve) but also in more typical cases.

We begin by noting that a completely uninformed predictor (e.g., flip a weighted coin to determine the prediction) will have the parametric form s=q. This is because such predictor will only need to be consistent with the rate of occurrence of extreme events. Any other predictor will have s>q at least somewhere. Note that if we find a predictor with s<q this can be transformed into a good one by just taking the opposite guess. To this end, it is meaningful to consider the difference between the AUC of any arbitrary predictor, α(q), and the completely uninformed one which has AUC equal to *q*. That is, we should take the amount by which the predictor is better than an appropriately weighted coin, in the sense of area under the precision–recall curve. Moreover, α(q) will vary in value as *q* is varied. Our extreme event predictor should single out one specific threshold that corresponds to the most effective separation (between extremes and quiescent events), the threshold that corresponds to the best predictive power.

Based on this analysis, we suggest the maximum adjusted area under the curve, $\alpha^{*}$, as a choice of metric, given by

(17) $$\alpha^{*}=\max_{q\in [0,1]}\left(\alpha \left(q\right)-q\right).$$

The quantity α(q)−q is a measure, at extreme event rate *q*, of how much better a predictor *B* is than the uninformed predictor. When α(q)−q≫0, the predictor does an excellent job of predicting extreme events at the threshold $\hat{a}$ corresponding to the extreme event rate *q*. Conversely, when α(q)−q≈0 (or even α(q)−q<0), the predictor is poor at that extreme event rate. Below, we examine several analytical forms of joint pdfs between indicators and predictors, to understand the mechanics of the volume under the surface metric and the maximum adjusted area under the curve.

### 4.1. Coinflip Indicator—Predictor

**Figure 5:**
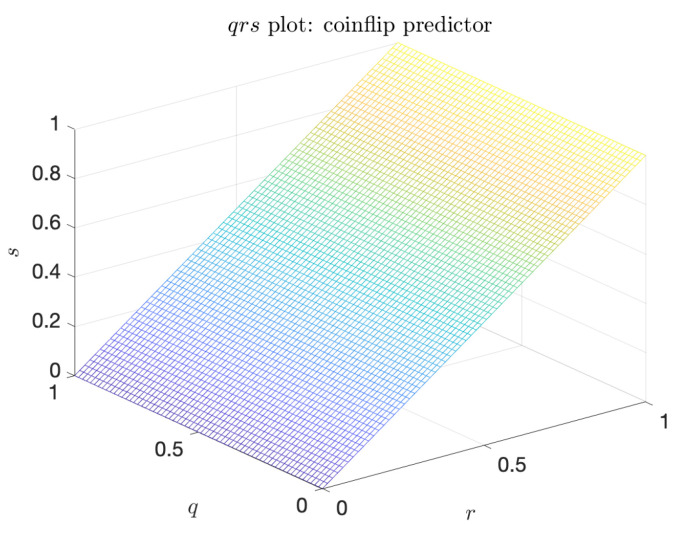
Plot of the qrs surface for the coinflip predictor. V=0.5, $\alpha^{*}=0$.

The coinflip predictor is the naive predictor that is completely independent of the indicator. Its characteristic triangular shape, shown in Figure 5, has the minimum value of the volume under the surface: V=0.5. Further, the coinflip predictor is the baseline against which the metric $\alpha^{*}$ is constructed. Indeed, for the coinflip predictor, $\alpha^{*}=0$.

### 4.2. Bimodal Indicator—Predictor

A repository with sample codes for the following subsections may be found on Github. Sample implementations of the algorithms discussed may be found at https://doi.org/10.5281/zenodo.3417076, or https://github.com/batsteve/ferocious-cucumber. To demonstrate the relationship between separation of data (probability bimodality) and $\alpha^{*}$, we consider a bimodal pdf, given by:

(18) $$f_{ab}\left(a,b;\gamma ,\rho \right)=\frac{1}{\beta }\left[e^{-\left(a^{2}+b^{2}\right)\rho^{2}}+\gamma e^{-\left({\left(a-1\right)}^{2}+{\left(b-1\right)}^{2}\right)\rho^{2}}\right]$$

**Figure 6:**
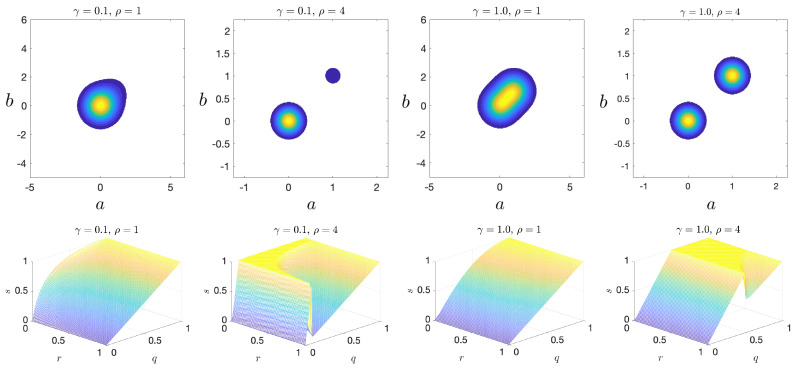
(**top**) Joint pdf plots of the bimodal scenario for various parameters; and (**bottom**) corresponding qrs plots.

This function is the sum of two Gaussian modes: a quiescent mode centered at (0,0) and an extreme event mode centered at (1,1). The pdf is further controlled by two parameters: γ and ρ. The parameter γ controls the weight of the extreme mode relative to the quiescent mode, and ρ controls the spacing of the modes. Figure 6 shows the joint pdf and the corresponding qrs plots for representative parameter values. Note that the knuckle becomes more pronounced as ρ (separation) increases.

**Figure 7:**
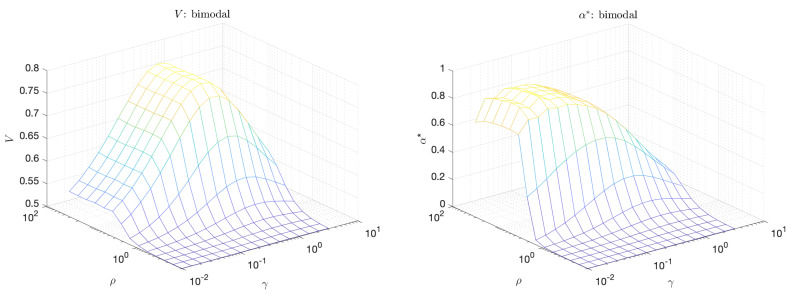
Volume under the curve (*V*) (**left**); and maximum adjusted area under the curve ($\alpha^{*}$) (**right**) for the bimodal scenario.

The plots in Figure 7 shows the volume under the curve, *V*, and the maximum adjusted area under the curve, $\alpha^{*}$, for the bimodal scenario as a function of γ and ρ. We note that *V* is largest when the separation between extreme and quiescent events, ρ, is large but γ is close to 1 (equal distribution of probability mass between extreme and and quiescent events). On the other hand, $\alpha^{*}$ peaks even at small values of γ, a scenario which is more realistic for extreme events occurring in physical systems due to internal instabilities, and which cannot be captured with the standard approach relying on *V*. This clearly demonstrates the advantage of the introduced criterion for the selection of optimal predictors.

### 4.3. Gaussian Indicator—Predictor

**Figure 8:**
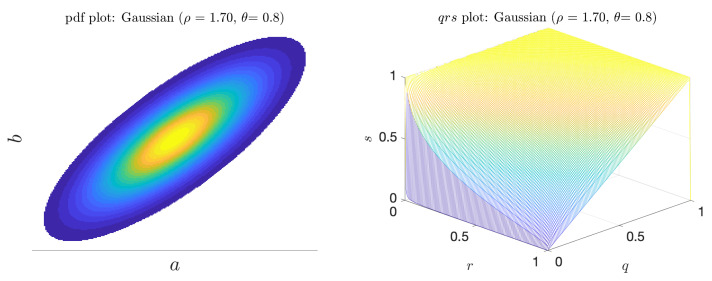
Joint pdf plot (**left**); and the corresponding qrs surface for the Gaussian scenario (**right**).

**Figure 9:**
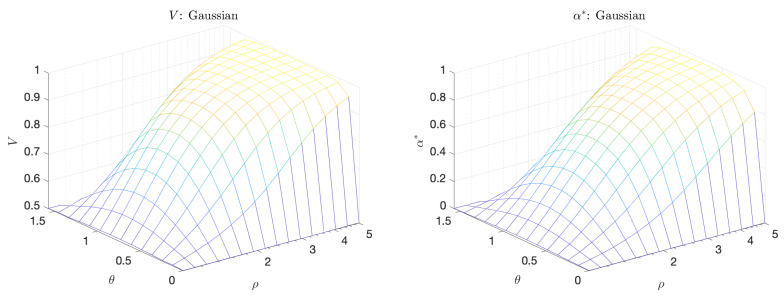
Volume Under the Curve (*V*) (**left**) and Maximum Adjusted Area Under the Curve ($\alpha^{*}$) (**right**) for the Gaussian scenario.

The multivariate Gaussian scenario is described by a pdf of the form

(19) $$f_{ab}\left(a,b;\rho ,\theta \right)=\frac{1}{\beta }e^{-{\left(\cos\theta a+\sin\theta b\right)}^{2}\frac{1}{\rho^{2}}-{\left(\sin\theta a-\cos\theta b\right)}^{2}\rho^{2}}$$

where β is a normalization factor, $\rho^{2}$ is the length ratio of the two principal axes, and θ is the angle between the principal axis and the *a* axis. Figure 8 shows a sample pdf. When θ=0 or $\theta =\frac{\pi }{2}$, there is no linear (or higher order) correlation between the indicator and predictor and in both cases the predictor is merely the coinflip predictor. However, when θ is near $\frac{\pi }{4}$, and ρ>1, there *is* a (linear) correlation between *a* and *b*. In this regime, we see both *V* and $\alpha^{*}$ increase (Figure 9). Unlike the bimodal scenario, there is no distinct scale separation. As a result, *V* and $\alpha^{*}$ track each other closely.

## 5. Applications

### 5.1. The Majda–McLaughlin–Tabak (MMT) Model

The Majda–McLaughlin–Tabak (MMT) model is a 1D nonlinear model of deep water wave dispersion first introduced in [23], and since studied in the context of weak turbulence and intermittent extreme events [25,28,29]. The governing equation is given by

(20) $$iu_{t}=|\partial_{x}{|}^{\alpha }u+\lambda {\left|\partial_{x}\right|}^{\frac{-\beta }{4}}\left(\left|\right|\partial_{x}{|}^{\frac{-\beta }{4}}u{|}^{2}{\left|\partial_{x}\right|}^{\frac{-\beta }{4}}u\right)+iDu,$$

where *u* is a complex scalar, while the pseudodifferential operator $|\partial_{x}{|}^{\alpha }$ is defined through the Fourier transform as follows:

$$\hat{|\partial_{x}{|}^{\alpha }u\left(k\right)}={\left|k\right|}^{\alpha }\hat{u(k)}.$$

Here, we select the system parameters following the authors of [25], who employed the MMT equation to validate a model for nonlinear wave collapse and extreme event prediction. The domain has spatial extent 2π, discretized into 8192 points, and temporal extent 150, discretized into 6000 points for integration purposes. The parameters are chosen so that λ=−4 (focusing case), $\alpha =\frac{1}{2}$ (deep water waves case), and β=0. The operator Du is a selective Laplacian designed to model dissipation at small scales, i.e., due to wave breaking. The first 1000 points are discarded to avoid transients due to random initial conditions; no forcing term is included and the simulations represent free decay.

Extreme events are indicated by large values of the wave group amplitude:

(21) $$A\left(x_{0},t_{0}\right)=\left|u\left(x_{0},t_{0}\right)\right|.$$

**Figure 10:**
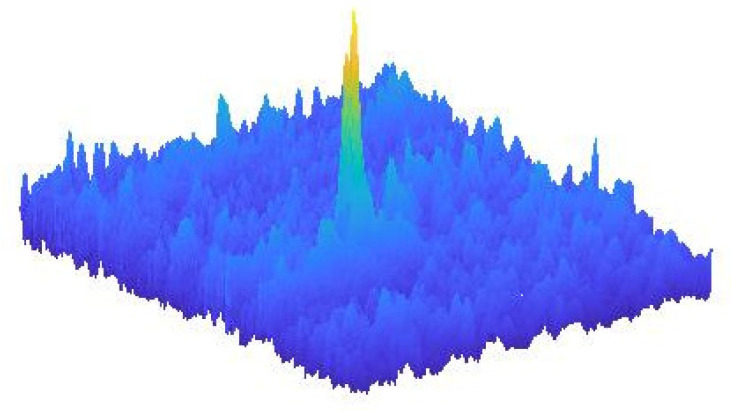
Sample plot of one simulated realization of the Majda–McLaughlin–Tabak (MMT) model near an extreme event.

Figure 10 shows sample realization of the MMT model near an extreme event.

**Figure 11:**
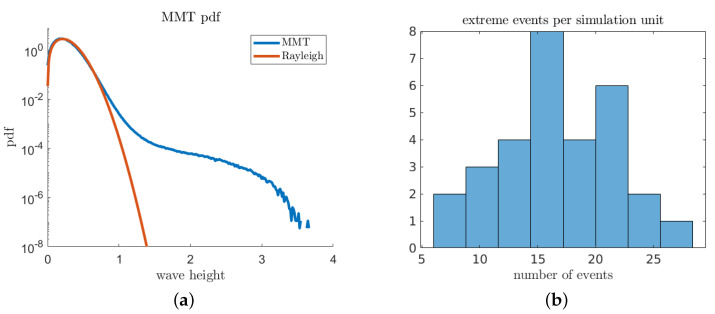
(**a**) Probability density function of the MMT wave height. Rayleigh distribution overlaid for comparison (note the “long-tail’ extending from x≈1.5 to x≈3.5); and (**b**) histogram of the number of extreme events for different simulation runs.

We construct predictors using a simple machine learning paradigm: surrogate optimization across a specified hypothesis space. We compare $\alpha^{*}$ (introduced in Equation (17)) against other standard binary classification objectives, such as $F_{1}$-score and total accuracy. For total accuracy and $F_{1}$-score, we use an extreme-event threshold $\hat{a}=1.5$ (Figure 11). The hypothesis class for the predictor is chosen as a two-element linear combination of *k* zero-order Gabor coefficients of variable length scales [30]:

(22) $$h_{P}\left(x\right)=a_{1}\exp\left(-\frac{x^{2}}{2L_{1}^{2}}\right)+a_{2}\exp\left(-\frac{x^{2}}{2L_{2}^{2}}\right).$$

These functions can be conceptualized as localized Gaussian wavelets. We use a standard implementation of a surrogate search [31,32] to identify the optimal values for $\frac{a_{1}}{a_{2}},L_{1},L_{2}$. Except where otherwise noted, we terminated optimization after 100 function evaluations when calculating from 1 unit of simulation data.

In the MMT model, extreme events are localized both spatially and temporally. To convert from simulation data into training pairs, we use strict time-lags and spatial maxima. The rule strict time-lag means that a prediction from time $t_{0}$ is always paired to an indicator measured at exactly time $t_{0}+\tau$. This underestimates predictor quality by throwing out “near-misses,” but avoids the definitional quagmire needed to avoid the issue. The spatial maximization rule means that the most extreme prediction anywhere (at fixed time $t_{0}$) is compared to the most extreme event anywhere (at fixed time $t_{0}+\tau$). This overestimates goodness by including “false coincidences”, however, in simulation, false coincidences are extremely rare.

#### Numerical Results

Regardless of the objective function, all the computed optimal predictors are typically characterized by a short length scale component, a long length scale component, and an amplitude weighting greatly favoring the short component. This breakdown has a simple physical interpretation that agrees with with previous work [25]. In order for an extreme event to occur, there must be sufficient background energy to draw up (long length scale), and also enough localized “seed” energy which will begin the collapse.

**Figure 12:**
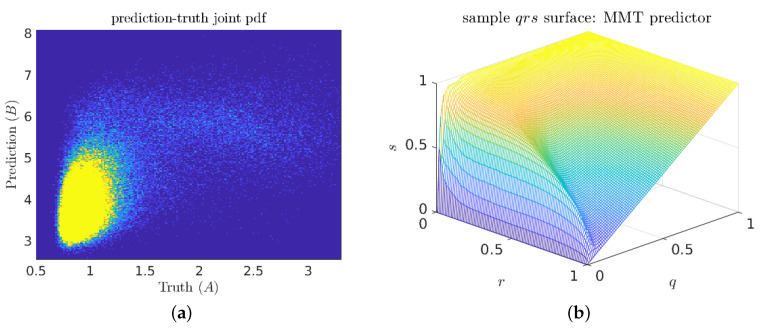
(**a**) Sample prediction–truth joint pdf for a good MMT predictor; and (**b**) corresponding qrs surface plot.

Exploratory investigations of three-component predictors (with five adjustable parameters) almost invariably collapsed onto two-vector solutions. This suggests that the two length-scale interpretation of the optimal predictors is not just a necessary artifact of hypothesis space dimension. The joint prediction–truth pdf (Figure 12a) does not appear to contain any scale separation. Indeed, the only easily visible feature is the density peak in the low-prediction low-indicator corner (true negatives). However, in the associated qrs surface plot (Figure 12b), the knuckle feature near r=0.05 suggests that there is some hidden scale separation.

**Figure 13:**
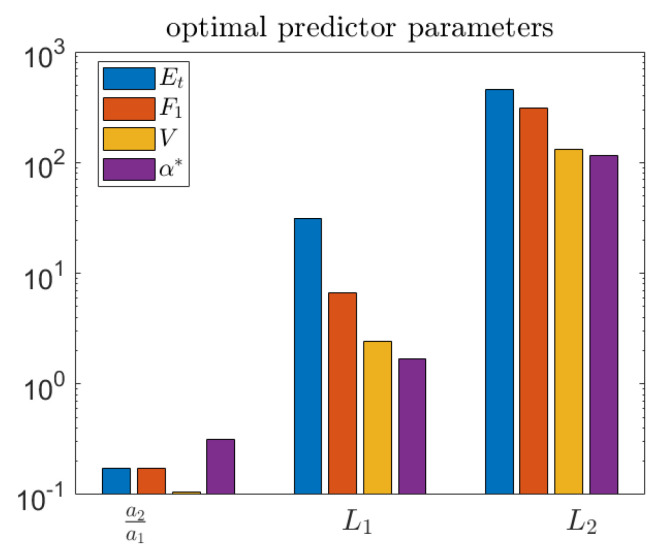
Optimal predictor parameters for each objective function. Note that total accuracy is very different from the others.

The different choices of objective function lead to different optimal parameters, as shown in Figure 13. Three of the objectives, $F_{1}$, *V*, and $\alpha^{*}$, result in similar parameter values, while the total accuracy criterion, $E_{T}$, is quite different—generally resulting in longer length scales for the predictor.

**Figure 14:**
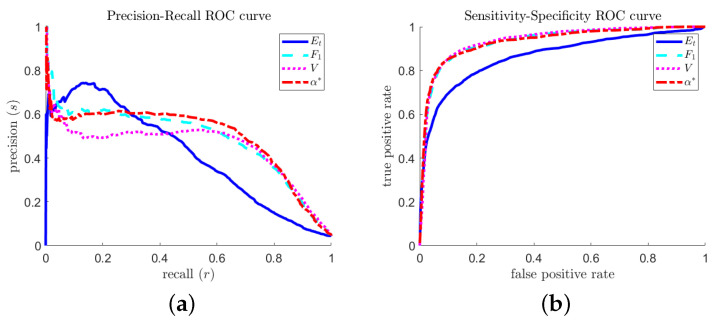
Receiver operating characteristic curve comparisons of optimal predictors calculated via different objectives: (**a**) precision–recall curve; and (**b**) sensitivity–specificity curve.

A natural question is: How do the predictions (of the different optimal predictors) differ, and which one is better? Figure 14 shows two sets of ROC curves for the different optimal predictors. The precision–recall curve (Figure 14a) shows that the total-accuracy-optimized predictor can achieve slightly better precision at very low recall tolerances, but otherwise performs more poorly. In typical extreme event prediction contexts, a high recall (avoid false negatives) is a more valuable property.

The near-50% precision rate (Figure 14a) reflects an approximate temporal symmetry of the MMT extreme event mechanism: focusing and defocusing energy distributions look very similar, especially within a restricted hypothesis space that discards phase information.

**Figure 15:**
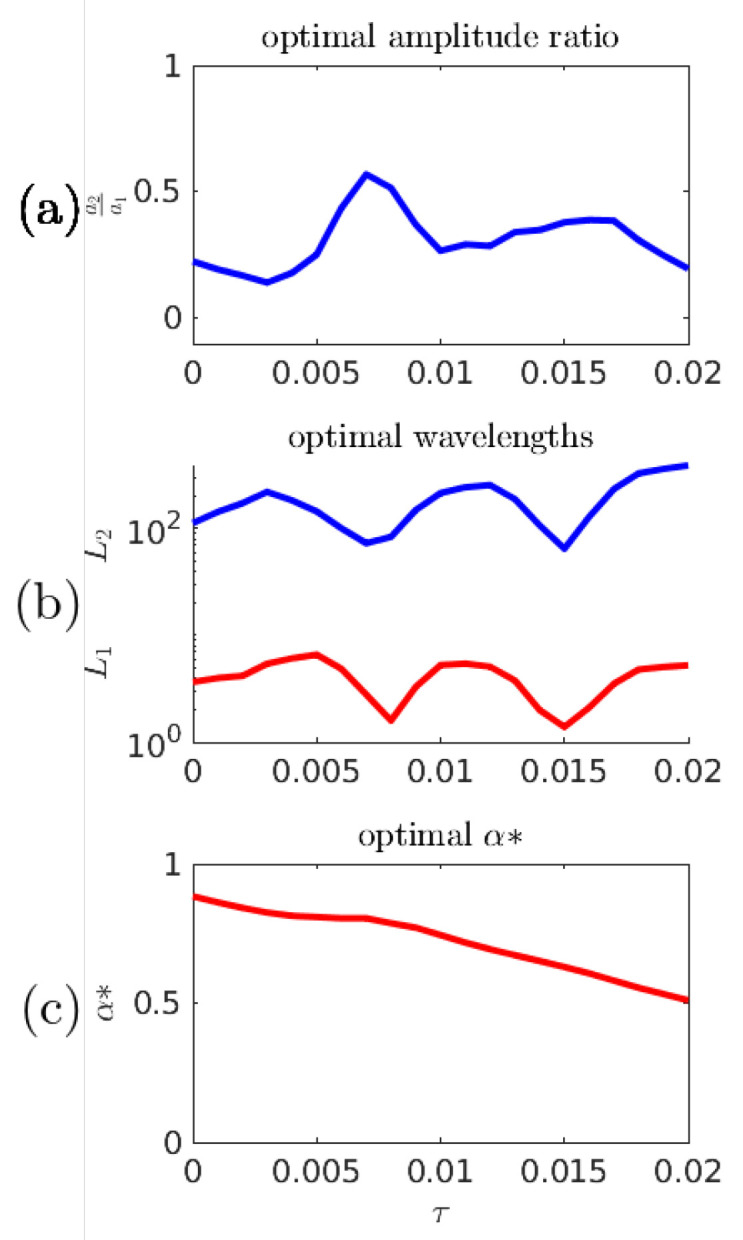
(**a**,**b**) Optimal predictor parameters as a function of τ; and (**c**) optimal $\alpha^{*}$ as a function of τ.

In the previous numerical experiments, a fixed time gap τ=0.015 has been used to represent a suitable prediction time scale: long enough for significant wave evolution, short enough that good predictions are better than blind chance. Figure 15 shows the optimal predictor parameters associated with the $\alpha^{*}$ objective function for other choices of τ. The dramatic drop near τ=0.007 obscures the fact that the predictor parameters are interdependent–the increase of the amplitude ratio partially counteracts the effects of the shorter length scales.

### 5.2. The Kolmogorov Flow Model

The Kolomogorov flow is a solution to the forced Navier–Stokes problem on a 2D periodic domain. Above Re≈35, the solution is unstable, and there are intermittent bursts of energy dissipation [14]. The Navier–Stokes equations (pressure–velocity form), defined over some domain $\Omega \in R^{2}$, are given by

(23) $$\begin{array}{cc} \partial_{t}u & =-u\cdot \nabla u-\nabla p+\nu \Delta u+f\\ \nabla \cdot u & =0 \end{array}$$

where *u* is the (vector valued) fluid velocity field, *p* is the (scalar valued) pressure field, ν is the dimensionless viscosity (inversely related to the famous Reynolds number) and *f* is some forcing term. In the Kolmogorov flow model, the forcing is a monochromatic time invariant field given by

(24) $$f\left(x\right)=\sin\left(k_{y}\cdot x\right){\hat{e}}_{1}$$

where $k_{y}=\left(0,4\right)$ is the wavenumber of the forcing field and ${\hat{e}}_{1}=\left(1,0\right)$ is a unit vector perpendicular to $k_{y}$. The intermittent bursting phenomena associated with the Kolmogorov flow for large enough Reynolds numbers (Re⪆35) are captured by the energy dissipation rate, given by

(25) $$E_{diss}\left(u\right)=\frac{\nu }{\left|\Omega \right|}\int_{\Omega }{\left|\nabla u\right|}^{2}dx$$

**Figure 16:**
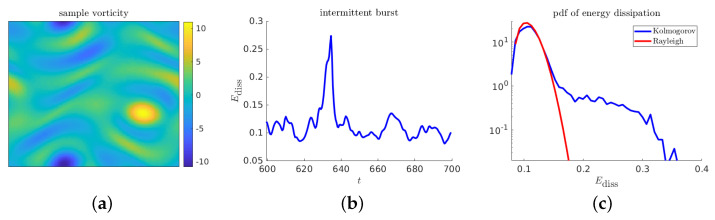
Descriptive plots for the Kolmogorov flow: (**a**) sample realization of the the vorticity; (**b**) time series of energy dissipation near an extreme event; and (**c**) pdf of the energy dissipation.

Figure 16 contains descriptive plots for the Kolmogorov flow model: two visualizations of a sample realization, and the pdf function.

A natural global set of predictors are the coefficients associated with low-*k* 2D Fourier modes, $b_{k}$. We also consider arbitrary linear combinations of these coefficients, that is, predictors given by

(26) $$B=\Sigma_{k}\gamma_{k}b_{k}$$

Other machine learning algorithmic choices closely track those employed for the MMT model, although intermittent events are spatially global so there is no spatial maximization required.

#### Numerical Results

**Figure 17:**
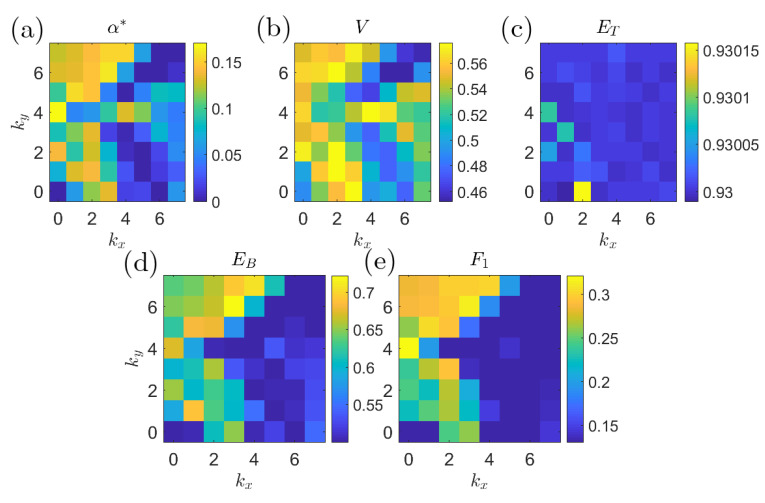
Plots of single coefficient predictor quality for different wavenumbers and objectives: (**a**) $\alpha^{*}$; (**b**) volume under the surface; (**c**) total accuracy; (**d**) balanced error; and (**e**) $F_{1}$ score. Note the consistent peak at (0,4), which is resolved best by $\alpha^{*}$ and $F_{1}$.

**Figure 18:**
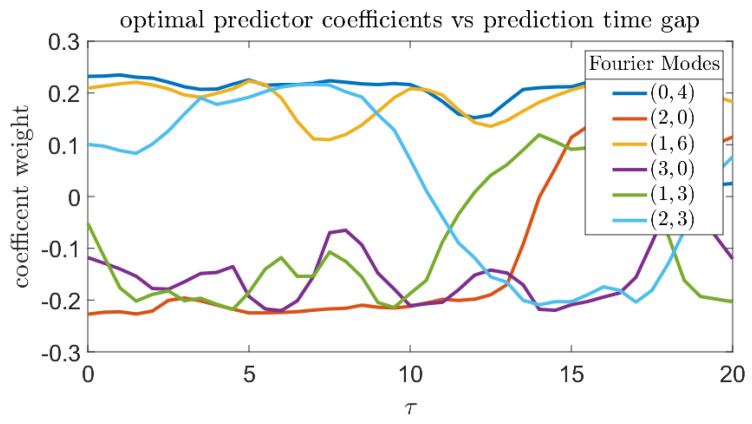
Composition of optimal predictor, in terms of Fourier modes as a function of prediction gap τ. Positive coefficients correspond to increasing extreme event likelihood, while negative coefficients correspond to suppression.

Figure 17 shows the prediction quality of the single-coefficient predictors according to different metrics. By every metric, the Fourier coefficient with wavenumber k=(0,4) has consistently good predictive properties. This is consistent with the findings in [14] where a variational approach was employed to compute precursors directly from the governing equations and some basic properties of the system attractor. Figure 18 plots the six most significant $\gamma_{k}$ from Equation (26) to show the effects of changing τ on the composition of the optimal predictors.

The next numerical study involves optimization of a combined predictor employing several wavenumbers. Even in this case, the most important component from the combined predictor is the (0,4) mode, and its weighting factor is always positive. Other Fourier modes, such as (3,0), have a negative weighting factor, which means they are inversely correlated with bursts of extreme dissipation. Due to the consistent downward trend in prediction quality as τ increases, trends in the data past τ≈15 are less likely to be meaningful.

## 6. Conclusions

We have formulated a method for optimizing extreme event prediction in an equation free manner, i.e., using only data. Our first aim was to understand critical limitations of binary classification methods in the context of precursors for extreme events. We then showed how the qrs surface construction allows for a geometric interpretation of scale separation, and naturally led to the metric $\alpha^{*}$, which is well suited to this problem. We compared $\alpha^{*}$ to other metrics in two models of extreme events, where we showed that $\alpha^{*}$ selects for qualitatively better predictions than the total accuracy, and has superior optimization properties as compared to $F_{1}$-score.

While the main focus of this work was a way to construct a prediction metric suited to the problem of extreme event prediction, there are still important questions related to the selection of good hypothesis classes, and the binning procedure to go from trajectory data to training pairs. While we believe both these questions are highly problem dependent, any attempt to apply the machine learning paradigm to a related problem must focus on these issues carefully.
